# Facilitating duodenoscope insertion with a balloon overtube in a patient with gastric deformity

**DOI:** 10.1055/a-2584-1901

**Published:** 2025-05-06

**Authors:** Yosuke Ohashi, Takuji Iwashita, Shota Iwata, Shinya Uemura, Masahito Shimizu

**Affiliations:** 1 476117 First Department of Internal Medicine, Gifu University Hospital, Gifu, Japan

The side-viewing feature of duodenoscopes, which are dedicated endoscopes for endoscopic retrograde cholangiopancreatography (ERCP), can occasionally complicate endoscopic insertion, particularly in patients with gastric deformity or tumor infiltration. We report a case in which balloon overtube placement facilitated duodenoscope insertion in a patient with gastric deformity.

An 86-year-old man with malignant biliary obstruction secondary to pancreatic cancer had previously undergone transpapillary placement of a covered self-expandable metallic stent (cSEMS). The patient presented with cholangitis, and computed tomography (CT) confirmed biliary dilation suggestive of recurrent stent occlusion ( Fig. 1 ). ERCP with stent exchange was planned. However, duodenoscope insertion was unsuccessful due to gastric deformity, likely caused by tumor invasion. Therefore, the duodenoscope was exchanged for a balloon endoscope (SIF-Q260, Olympus) with a balloon overtube (ST-CB1, Olympus) with an inner diameter of 13.8 mm and a length of 77 cm. The forward-viewing endoscope enabled a successful insertion into the duodenum. The balloon overtube was then advanced into the gastric antrum ( Fig. 2 ), and the balloon was inflated to secure its position. The endoscope was removed while maintaining overtube placement. Subsequently, a duodenoscope with an outer diameter of 12.9 mm (JF-240, Olympus) was successfully inserted through the overtube ( Fig. 3 , Video 1 ). Stent exchange with a 6 mm diameter cSEMS was then performed without complications ( Fig. 4 ).

**Fig. 1:**
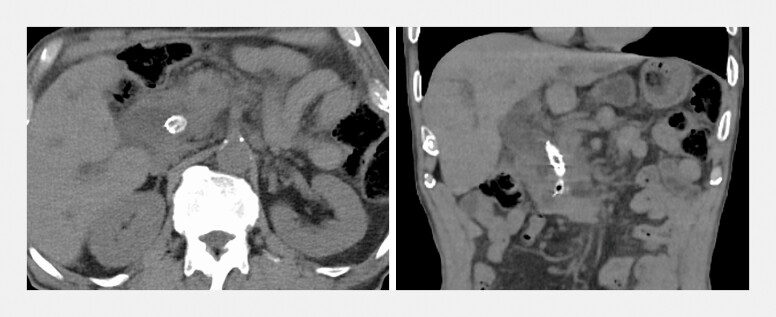
Computed tomography image showed dilation of the bile duct due to the stent occlusion.

**Fig. 2:**
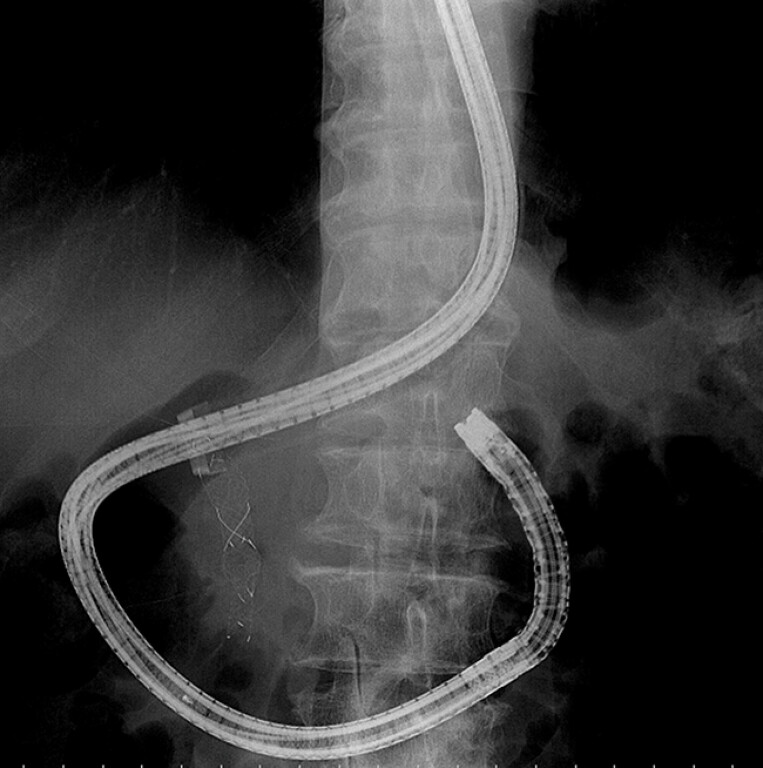
The balloon overtube was then advanced into the gastric antrum.

**Fig. 3:**
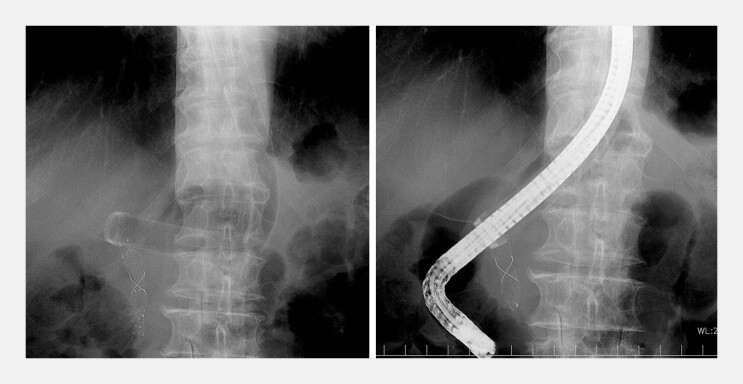
The duodenoscope was inserted through the overtube.

**Fig. 4:**
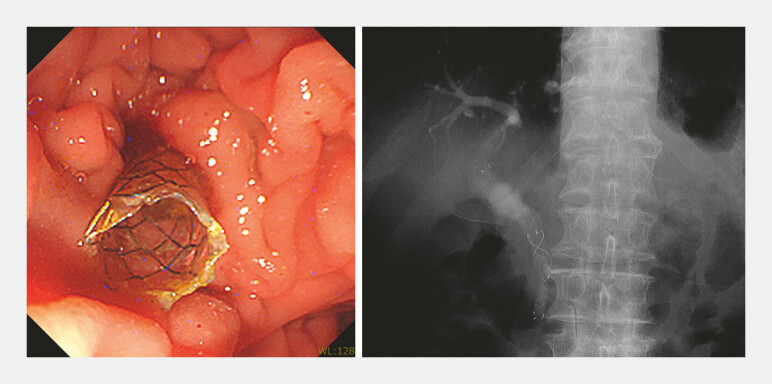
Stent exchange with a 6 mm diameter covered self-expandable metallic stent was performed.

A duodenoscope with an outer diameter of 12.9 mm was inserted through the overtube.Video 1

Several techniques have been reported to facilitate duodenoscope insertion, including guiding technique using a guidewire and catheter for esophageal insertion 1 , anchoring technique by a dilation balloon or basket catheter for stricture 2 3 , and application of overture to increase pushability of the endoscope for perigastric adhesions 4 . Regarding through overtube techniques, esophageal insertion using a short overtube has been reported to manage the pharyngeal pouch 5 . This duodenoscope insertion technique using the balloon overtube offers a valuable option for overcoming upper gastrointestinal obstacles, although the overtube inner diameter may limit duodenoscope selection.

Endoscopy_UCTN_Code_TTT_1AR_2AB

## Notes

Endoscopy E-Videos https://eref.thieme.de/e-videos
*E-Videos* is an open access online section of the journal *Endoscopy* , reporting on interesting cases and new techniques in gastroenterological endoscopy. All papers include a high-quality video and are published with a Creative Commons CC-BY license. Endoscopy E-Videos qualify for HINARI discounts and waivers and eligibility is automatically checked during the submission process. We grant 100% waivers to articles whose corresponding authors are based in Group A countries and 50% waivers to those who are based in Group B countries as classified by Research4Life (see: https://www.research4life.org/access/eligibility/ ). This section has its own submission website at https://mc.manuscriptcentral.com/e-videos .

## References

[1] Wai CT Yeoh KG Ho KY Esophageal intubation with duodenoscope in the presence of pharyngeal pouch by a guidewire and catheter-guided technique Surg Laparosc Endosc Percutan Tech 2002 12 362 363 10.1097/00129689-200210000-00012 12409706

[2] Kikuyama M Itoi T Sasada Y Large-balloon technique for one-step endoscopic biliary stenting in patients with an inaccessible major papilla owing to difficult duodenal stricture (with video) Gastrointest Endosc 2009 70 568 572 10.1016/j.gie.2009.03.032 19573866

[3] Kawakami H Kubota Y Ban T A duodenoscope anchoring technique in a case of difficult scope intubation due to scope-pyloric ring misalignment Endoscopy 2021 53 E455 E456 10.1055/a-1327-1813 33540436

[4] Kawakami H Uchiyama N Ogawa S Duodenoscope insertion difficulty due to perigastric adhesions overcome using an overtube J Hepatobiliary Pancreat Sci 2023 30 e84 e85 10.1002/jhbp.1362 37792664

[5] Dickey W Porter KG Duodenoscope intubation of the oesophagus in the presence of pharyngeal pouch made possible by an overtube Endoscopy 1995 27 212 213 10.1055/s-2007-1005667 7601058

